# A misaligned magneto-optical trap to enable miniaturized atom chip systems

**DOI:** 10.1038/s41598-018-28464-0

**Published:** 2018-07-04

**Authors:** Ritayan Roy, Jo Rushton, Andrei Dragomir, Matthew Aldous, Matt Himsworth

**Affiliations:** 1 School of Physics and Astronomy, University of Southampton, Highfield, Southampton, SO17 1BJ United Kingdom; 2 University of Birmingham/National Physical Laboratory, Hampton Rd, Teddington, Middlesex TW11 0LW UK; 3 Present Address: School of Mathematical and Physical Sciences, University of Sussex, Falmer Campus, Brighton, BN1 9QH United Kingdom

## Abstract

We describe the application of displaced, or misaligned, beams in a mirror-based magneto-optical trap (MOT) to enable portable and miniaturized atom chip experiments where optical access is limited to a single window. Two different geometries of beam displacement are investigated: a variation on the well-known ‘vortex-MOT’, and the other a novel ‘hybrid-MOT’ combining Zeeman-shifted and purely optical scattering force components. The beam geometry is obtained similar to the mirror-MOT, using a planar mirror surface but with a different magnetic field geometry more suited to planar systems. Using these techniques, we have trapped around 6 × 10^6^ and 26 × 10^6^ atoms of ^85^Rb in the vortex-MOT and hybrid-MOT respectively. For the vortex-MOT the atoms are directly cooled well below the Doppler temperature without any additional sub-Doppler cooling stage, whereas the temperature of the hybrid-MOT has been measured slightly above the Doppler temperature limit. In both cases the attained lower temperature ensures the quantum behaviour of the trapped atoms required for the applications of portable quantum sensors and many others.

## Introduction

Progress in science and technology over the last decades has shown that miniaturization and integration can lead to robust applications of fundamental physics, be it the miniaturization of electronics by integrated circuits or in optics in terms of micro-optical devices and sensors. In atomic physics, atom chip experiments based on neutral atoms or ions are starting to be realized in similar scalable quantum-optical systems. An atom chip, at its most basic, is a substrate with microfabricated conductors which produce magnetic and electric fields that can be used to trap and manipulate atoms and ions. Atom chips enable highly sophisticated experiments to be condensed into areas on the order of a few square centimetres and readily lend themselves to the miniaturization and integration of cold atom systems for practical applications beyond the laboratory.

The concept of creation of magnetic traps using micro-structured chips was proposed by Weinstein *et al*.^1^. There followed developments with discrete wires^2–6^ and permanent magnets^7–10^, but the first successful realization of a trap on an atom chip is by Reichel *et al*.^11^ and Folman *et al*.^12^ using a technique called the mirror-MOT. The beam geometry of the standard mirror-MOT is not ideal for future microfabricated vacuum cells which have limited optical access due to the beam propagating parallel to the mirror surface^13,14^. There are various schemes to create a MOT within small volumes^15–18^, however they require complex and expensive microfabrication procedures and several of these designs are not easily compatible with planar atom chip structures.

**Figure 1:**
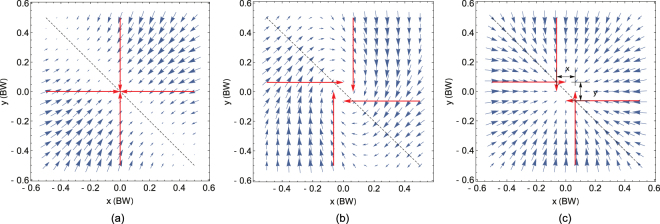
Analytical simulations of the restoring force vectors (blue arrows) on a stationary atom in the *xy* plane. The axes are in units of (1/*e*^2^) beamwidth. The red arrows indicate the direction of the beams incident at 45° along the *z* axis out of the page. The tips of the red arrows signify the point of reflection on the mirror surface. In (**a**) there is no misalignment resulting in no restoring force along the diagonal highlighted with a dashed line. By misaligning the beams shown in (**b**) the beams produce a vortex-like restoring force, whereas swapping the beams propagating in the *y* direction results in the ‘hybrid’ restoring force (polarizations remain unaltered). The separations between beams along the *x* and *y* axes the beams are Δ_*x*_ and Δ_*y*_, respectively and are always symmetrical along the line of zero magnetic field at *x* = −*y* = 0.

Recently, to solve the above limitations, a novel switching-MOT technique (S-MOT)^19^ was reported which relies on the synchronized dynamic switching of magnetic and optical fields. In this scheme, the magnetic fields are produced solely from conductors in a single plane, and all beams are incident on the mirror at 45° thus only requiring a single viewport. To simplify the S-MOT technique further, we have investigated how it would be possible to make a MOT without the switching of the optical and magnetic fields whilst retaining the same advantages. Here, we demonstrate two MOT techniques based on a DC magnetic field with displaced MOT beams which are referred to as the *vortex*-*MOT* and *hybrid*-*MOT*. The vortex-MOT, which is formed by displacing the MOT beams in a vortex pattern (see Figs 1(b) and 3(d)), is in a similar vein to an earlier demonstration^20^ but adopted for a single optical window. In contrast, the novel hybrid-MOT (Figs 1(c) and 3(d)) technique is created by an elegant balancing between the trapping forces created via pure optical scattering and Zeeman-shifted resonances.

## Theory

The S-MOT is based around a quasi-quadrupole magnetic field produced by two orthogonal pairs of separated conducting wires. The aim is to find an alternative planar geometry, other than anti-Helmholtz coils, to produce the correct field gradients, in much the same manner as in Wildermuth *et al*.^21^, but avoiding any external bias fields. The lack of rotation symmetry of this geometry resulted in an axis over which no trapping will occur, as shown in Fig. 1(a), and so our effort with the S-MOT, and this report, is to explore methods to close off this loss channel.

During experimentation of the S-MOT, it was noticed on several occasions that a dense cloud of atoms would form when all optical and magnetic fields are static and the beams are not perfectly aligned. Obtaining the cloud is surprisingly trivial as it would form quite readily, and significant effort was employed to avoid it whilst demonstrating the separate S-MOT mechanism. The range of operational parameters over which the static trap worked (alignment, polarization, beam intensity, detuning) ruled out a coherent argument for a single mechanism, but we believe most effects can be explained via two methods reported here. These trap geometries differ only via a small displacement on one pair of counter-propagating beams (see Fig. 1), however their principles of operation have a far greater disparity. We note that for both mechanisms, as well as the S-MOT, trapping and cooling in the *z* direction, orthogonal to the mirror, occurs via the usual (Zeeman-shifted) MOT mechanism, and we are only concerned here with obtaining trapping in the *xy* plane above the mirror.

## Vortex-MOT

In this geometry, shown in Fig. 1(b), each counter-propagating pair of beams are displaced by the same distance perpendicular from the incident plane, but in orthogonal directions. For example, the beam propagating in the [+*x*, +*z*] plane is displaced by a small distance along the +*y* axis, whereas the beam propagating along the [−*x*, +*z*] plane is displaced along −*y*. The resulting restoring force is not directed toward the trap center but instead follows a curl around the center - like a vortex - producing a torque on the atomic cloud. Traps operating in a similar manner, albeit not involving a mirror, have been demonstrated several times in the literature, and this mechanism is responsible for ‘race track’ patterns occasionally formed during the alignment of standard MOTs^22,23^. We will not discuss the theory here, as this has been thoroughly explored in the literature^24–26^. For trap parameters in which the cooling and restoring forces are greater than the vortex torque, the atoms will spiral in toward the trap center forming a spherical cloud. This is typically achieved when the beam separation, Δ, is less than the (1/*e*^2^) beamwidth, *ρ*, and is the parameter space this report explores. For weaker traps, greater atom number, and/or larger separations the cloud will begin to form rings around a small central cloud, or solely ring-shaped clouds.

## Hybrid-MOT

The hybrid mechanism is so-called as it encompasses both the usual Zeeman-shift dependent restoring force of the MOT (which we refer to as the ‘Zeeman’ force) along one direction, and the purely optical scattering force along the orthogonal direction (referred to as the ‘Optical’ force). In the latter case, by displacing the beams by a small amount symmetrically away from the trap center, an atom traversing the beam overlap volume experiences a variation in intensity between the two beams. The Gaussian beam-shape results in a local scattering minimum at the trap centre and results in a fully restoring force, as shown in Fig. 1(c). This mechanism was described in the earlier discussions within the literature of trapping via the scattering force^27^ and is related to the ‘two-beams-trap’ in which both the intensity and wavevector varies across the trap. To achieve a compact and spherical cloud of trapped atoms, the Optical and Zeeman force gradients must be balanced at the trap center. To find the optimum separation of the beams we simply model the Optical force as two equal and opposite Gaussian beams, with 1/*e*^2^ diameter, *ρ*, separated by Δ/2 from the trap center. Here, we shall use the [*u*, *v*] coordinate system to define the orthogonal axes in which the Zeeman and Optical force act independently to simplify the model. In relation to Fig. 1(c), the *u* axis corresponds to the diagonal marked by the dashed line where there is no magnetic field gradient, and *v* is orthogonal to this

1 $${F}_{s}(u)=\frac{\hslash k{\rm{\Gamma }}S}{2(1+{(\frac{2\delta }{{\rm{\Gamma }}})}^{2})}\,(\exp \,[\frac{\,-{(u+{\rm{\Delta }}/2)}^{2}}{(2{(\rho /4)}^{2})}]-\exp \,[\frac{\,-{(u-{\rm{\Delta }}/2)}^{2}}{(2{(\rho /4)}^{2})}])$$

where *k* is the beam wavevector, Γ is the natural linewidth, *δ* is the detuning from resonance (assumed negative for cooling), and *S* is the saturation power. This equation can be simplified to a linear function in *u*,

2 $${F}_{s}(u)=\frac{\hslash k{\rm{\Gamma }}S{\rm{\Delta }}|u|}{2{(\rho /4)}^{2}\,(1+{(\frac{2\delta }{{\rm{\Gamma }}})}^{2})}+O({u}^{2}).$$

**Figure 2:**
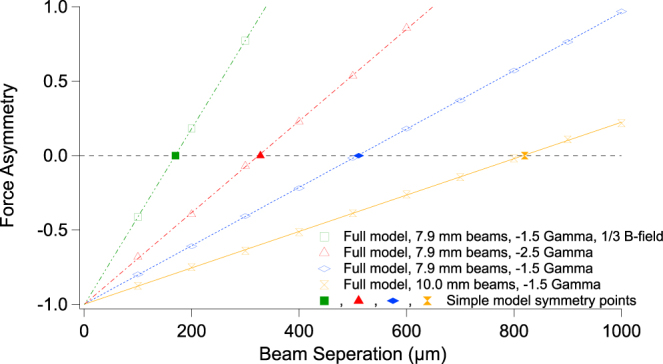
Comparison between the simple derivation (Equation 4, solid data points) and the full model (open data points) for the asymmetry of the trapping force in the hybrid MOT geometry as a function of beam separation with various trap parameters. The full model calculates the scattering force on a multilevel atom and accounts for saturation effects, beam parameters (shape, geometry and polarization) and an accurate simulation of the real magnetic field geometry. It does not assume a specific trapping mechanism and we have found it very reliable for a number of trap configurations. The plot calculates the asymmetry, *A* = (∂_*u*_*F* − ∂_*v*_*F*)/(∂_*u*_*F* + ∂_*v*_*F*) between the force gradients ∂_*u*,*v*_*F* along the *u* and *v* axes (diagonal to the *x*, *y* coordinate scheme). The simple model only predicts the optimum separation where the forces are equal and *A* = 0. We see remarkable agreement and further analysis confirms that the separation is independent of beam intensity. For an accurate prediction the magnetic field gradient used in Eq. 4 needs to be that found along the diagonal, not along the beam direction. At asymmetry values |*A*| ≥ 1 the trap is not stable.

The Zeeman force can be found in standard textbooks with a linear approximation (*S* < 1) of

3 $${F}_{z}(v)=\frac{\,-8kS\delta |v|}{{\rm{\Gamma }}{(1+{(\frac{2\delta }{{\rm{\Gamma }}})}^{2})}^{2}}g{\mu }_{B}|\frac{dB}{dv}|,$$

where g is the Land*é* g-factor, *μ*_*B*_ is the Bohr magneton, and $\tfrac{dB}{dv}$ is the magnetic field gradient. We have assumed throughout that the beam intensities do not saturate the transition, so that the beams can be treated independently, however we find that saturation effects are negligible for intensity-balanced beams. By equating equations 2 to 3 with |*u*| = |*v*|, and solving for Δ we find the optimum separation:

4 $${\rm{\Delta }}=\frac{-\alpha {\rho }^{2}\delta g{\mu }_{B}}{\hslash {{\rm{\Gamma }}}^{2}(1+{(\frac{2\delta }{{\rm{\Gamma }}})}^{2})}|\frac{dB}{dv}|,$$

where *α* is a geometrical factor which takes into account the increase in apparent beamwidth due to the incident angle and any variation in separation due to off-axis alignment. For this work in which the [*u*, *v*] axes are at 45° to the [*x*, *y*] axes, $\alpha =1/\sqrt{2}$, and the beamwidth along *u* remains unaltered from *ρ*. Equation 4 is valid for Δ ≤ |0.5*ρ*|, within which the Optical and Zeeman forces are approximately linear. As expected, this separation is highly dependent on beam width, as well as detuning, but independent of the beam intensity as both Optical and Zeeman forces are mediated by photon scattering and the saturation parameter, *S*, cancels out. We have confirmed this with a more detailed analytically model and have compared the prediction of equation 4 for a number of different trap parameters, such as detuning, beam size, and magnetic field gradients. The results are shown in Fig. 2. Independence from beam intensity is only true for near perfect balancing between counter-propagating beams. The full model does not assume any specific trapping mechanism other than the scattering force and was used to calculate the force patterns in Fig. 1, altering only the beam misalignments.

An important aspect to note is the beam displacements should always be perpendicular to the beam wavevector in the *xy* plane. From analytical simulations we have found that any misalignment along the wavevector plane causes an imbalance of beam intensity orthogonal to the mirror plane, *z*, thus shifting the trap center out of the beam overlap volume. This dependence also requires a high level of power balancing between each beam. The vortex-MOT is experimentally easier to produce as the atom cloud forms within a wide range of beam misalignments, even where the *x* and *y* displacement are not the same. The hybrid mechanism is far more sensitive to the beam separation. For example, in our system, the 7.9 mm diameter beams should be displaced by Δ = 0.5 mm, which is quite challenging, with a limit of trapping at Δ < 1 mm after which the Optical force overcomes the Zeeman force, as shown in Fig. 4. The sensitivity to misalignment increases with larger detunings and lower magnetic field gradients, and decreases for larger beams. Experimentally we expect the misalignment to slightly differ from the theoretical value due to intensity imbalances and polarization imperfections.

## Methods

**Figure 3:**
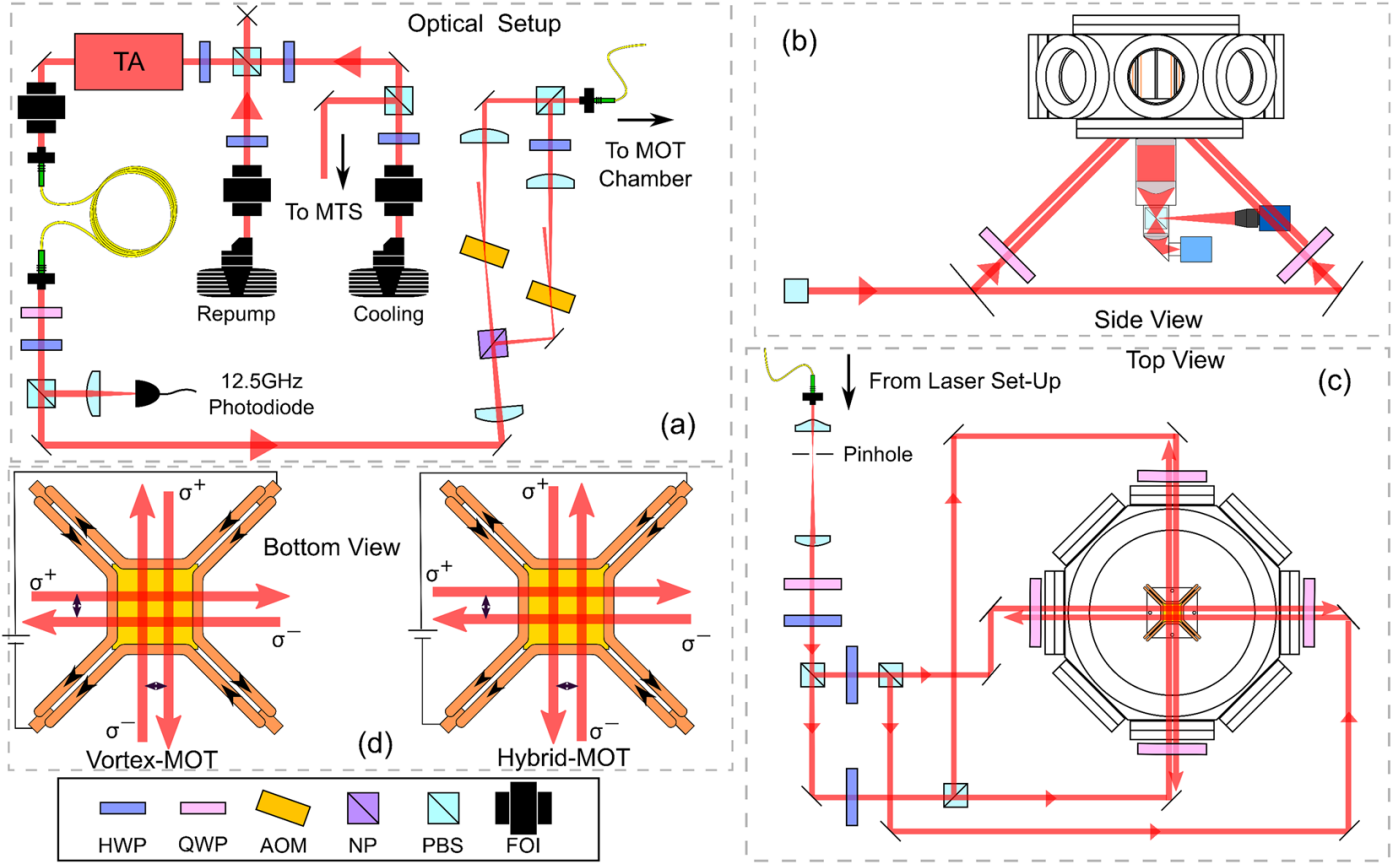
(**a**) The schematic of the optical setup used to generate the cooling and repump lasers for the MOT. (**b**) The side view of the vacuum chamber with imaging optics. The MOT geometries enables an increased imaging capability, as a large solid angle devoid of lasers is available for detection optics. For simplicity only one pairs of beams are shown. (**c**) The beams are cleaned with a pinhole and separated before being directed into the vacuum chamber. (**d**) A diagram of the wires used to generate the quadrupole field for the vortex-MOT and hybrid-MOT are shown, in addition to the beam displacements and their directions.

The laser setup for the experiment is shown in Fig. 3(a). A home-made external cavity diode laser (ECDL) provides the cooling light, which is frequency stabilized to the 5^2^S_1/2_, *F* = 3 → 5^2^P_3/2_, *F*′ = 4 cooling transition of ^85^Rb via modulation transfer spectroscopy (MTS)^28,29^. The beam from this cooling laser is combined with another beam from a similar ECDL, the repump laser. The combined beams are used to seed a tapered amplifier (TA, m2k TA-0785-2000-DHP), whose output is cleaned by passing through a single-mode fibre. A portion of the TA’s output is directed onto a fast photodiode (EOT ET-4000) and the resulting beat note is used to offset lock the repump from the cooling laser by the method described by J. Appel *et al*.^30^.

The cooling laser is detuned from the ^85^Rb cooling transition using acousto-optical modulators (AOMs). A detailed description of the optical setup is provided in the thesis of J. Rushton^31^. The combined cooling and repump beams are then sent to the MOT chamber using a non-polarization-maintaining optical fiber. At the other end of the fiber the beams are further cleaned with a spatial filter before being expanded to a 1/*e*^2^ diameter of *ρ* = 7.9. The beam is then distributed equally (in power) into four parts using three polarizing beam splitters and directed into the ultra-high vacuum (UHV) chamber with an incident angle of 45° to the mirror surface. The chamber has a single anti-reflection coated viewport and is maintained at a pressure of 2 × 10^−9^ mbar as measured from the lifetime of the trapped atoms^32^.

The quasi-quadrupole magnetic field is generated by two parallel pairs of wires, where both pairs carry current in the same direction and are situated inside the vacuum chamber. Each pair produces a two-dimensional approximation to a quadrupole magnetic field with a field zero equidistant between the wires. The addition of another perpendicular pair of wires does not produce a full 3D quadrupole magnetic field, only a 2D gradient rotated by 45° around the axis orthogonal to the mirror plane (see Fig. 1(a)). In effect, one could simplify the system further with a single pair wires to create the same field geometry.

An iris is used to reduce the beam diameter from 7.9 to 0.8 mm only for the alignment of the displaced beams. With the aid of fluorescence and the scattering of the beams from the mirror surface, the separation of the beams are measured to an accuracy of ±0.2 mm. To demonstrate the vortex-MOT, one pair of beams is displaced around Δ_*x*_ = 1.3 mm from the intensity maxima where as the other pair Δ_*y*_ = 1.0 mm. In the hybrid-MOT configuration, one pair of beams are separated by Δ_*x*_ = 0.7 mm and the other pair by Δ_*y*_ = 0.5 mm. The alignments are chosen to maximise the atom number and produce the most spherical cloud of atoms, hence the slight asymmetry, but agree with the theoretical optimum displacements within their uncertainty. We suspect these differences are due to slight intensity imbalances of the beams which can have a significant effect at differences of a few percent. The effect of the residual magnetic fields, such as the unshielded ion pump, is reduced with 3 external nulling coils.

With the atom cloud situated close to the viewport, a large solid angle devoid of lasers is possible for the fluorescence detection. Exploiting the favourable detection condition, as shown in the Fig. 3(b), we obtain a numerical aperture (NA) of ~0.6 using an aspheric condenser lens (Thorlabs ACL5040U-B). The details about the 2D quadrupole magnetic field generation is provided in the article^19^ and the whole experimental setup is summarized in Fig. 3. The quadrupole field gradient is estimated to be 9 G/cm along the *x* and *y* axes.

## Results

**Figure 4:**
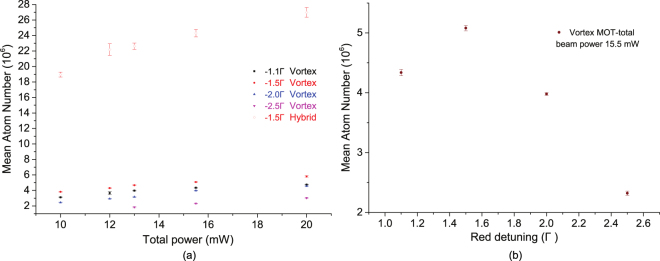
The number of trapped atoms for the vortex-MOT is measured as a function of the total MOT beam power and the red detuning of the cooling laser beams from the ^85^Rb cooling transition. For the hybrid-MOT the atom number are measured as a function of the total MOT beam power, keeping the optimum red detuning of the cooling laser beams at −1.5 Γ from the ^85^Rb cooling transition. (**a**) The mean atom number in the vortex and hybrid-MOT as a function of the MOT beam power. (**b**) The mean atom number of the vortex-MOT as a function of the cooling beam red-detuning.

**Figure 5:**
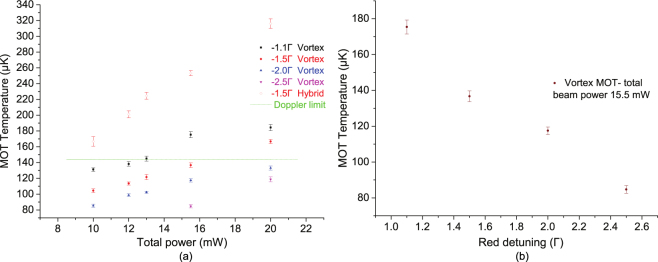
The temperature of the vortex-MOT is measured as a function of the total MOT beam power and the red detuning of the cooling laser from the ^85^Rb cooling transition. For the hybrid-MOT the temperature is measured as a function of the total MOT beam power, with optimum red detuning of −1.5 Γ from the ^85^Rb cooling transition. (**a**) An average temperature of the vortex and hybrid-MOT’s atom cloud as a function of the MOT beam power. The measured temperature is well below Doppler limit for the vortex-MOT, represented by green dashed line, without any additional sub-Doppler cooling stage. The measured temperature for the hybrid-MOT is slightly higher than the Doppler limit. (**b**) Temperature of the vortex-MOT atom cloud as a function of the of the cooling beam detuning.

In order to characterize the behaviour of the vortex-MOT, the temperature and the number of trapped atoms are measured as a function of the total MOT beam power and the detuning of the cooling laser from the ^85^Rb cooling transition. The total MOT beam power consists of the cooling and repump beams in 4.5:1 ratio respectively, which are equally distributed among four MOT beams at 45° to the mirror surface. For the hybrid-MOT the temperature and the atom number are measured only as a function of the total MOT beam power, keeping the detuning of the cooling laser fixed at −1.5 Γ from the ^85^Rb cooling transition. This is due to its sensitivity to alignments and detuning hence only a single configuration is used to compare with the vortex-MOT. The atom number and temperature measured for the vortex-MOT and hybrid-MOT are shown in the Figs 4 and 5 respectively, for comparison.

The atom number for both the MOTs are measured by the well known loading rate technique^32^. For this measurement, the camera (Fig. 3(b)) is replaced with a photodiode (Thorlabs PDA36A) for the detection of fluorescence signal. The vortex-MOT is loaded for around 3.3 seconds till the atom number is constant. The mean atom number of 10 such measurements represents a single datapoint in the Fig. 4(a). The maximum atom number of around 6 × 10^6^ is observed with a red-detuning of −1.5 Γ from the cooling transition and with the maximum available total power of 20 mW. We have repeated the hybrid-MOT atom number measurement same way as the vortex-MOT, but with the optimum detuning of −1.5 Γ from the cooling transition and the maximum atom number of around 26 × 10^6^ is observed. This was due to the difficulty in obtaining a stable spherical cloud in the hybrid configuration for other detunings. This agrees for the increased sensitivity to alignments and power imbalances as shown in Fig. 4, although lower detunings should significantly increase the optimum beam separation. The full model also predicts this effect but it was difficult to replicate experimentally. We suspect this to be due to instabilities and imperfections in the apparatus, such as interference fringes, when exploring this parameter space, and not the inaccuracies of the theory. The dependence of the atom number on cooling beam detuning for the vortex-MOT is shown in the Fig. 4(b). The vortex-MOT cloud size is consistently larger than hybrid-MOT, with average 1/*e*^2^ diameters of approximately 420 *μ*m and 380 *μ*m respectively. This results in a peak atom number density for the vortex-MOT of 1.5 × 10^11^ cm^−3^, and 9 × 10^11^ cm^−3^ for the hybrid-MOT.

The temperature of the atom cloud is measured using the time of flight (TOF) method. The beams are extinguished to allow the TOF for up to 5 ms and afterwards an imaging pulse illuminates the expanded cloud so it can be photographed by a fast CCD camera. After the image has been taken the cloud is allowed to disperse and then a second image is captured to be used for background subtraction. The TOF is limited by the geometry of the experimental setup and the imaging beam diameter. The width of the atom cloud after each time of flight is determined by a 2D Gaussian fit to the background-subtracted image. The temperature is determined by fitting a Gaussian distribution to the cloud image and the widths determined by the equation ${\sigma }^{2}={\sigma }_{0}^{2}+({k}_{{\rm{B}}}T/m){t}^{2}$, where *σ*_0_ and *σ* are the Gaussian widths before and after the expansion (TOF), *k*_B_ is the Boltzmann constant, *T* is the temperature of the cloud, *m* is the mass of ^85^Rb and *t* is the time of flight. Around 50 measurements are taken to determine an average temperature of the cloud. The temperature of the vortex and hybrid-MOT atom clouds as a function of the MOT beam power is presented in Fig. 5(a). Another plot in Fig. 5(b) represents the dependence of the temperature on the cooling beam red-detuning for the vortex-MOT. The vortex-MOT temperature reported here is well below the Doppler limit without any additional sub-Doppler cooling stage, whereas the measured temperature for the hybrid-MOT is slightly above.

## Discussion and Conclusion

Our results have shown that both the vortex and hybrid-MOT achieve characteristics similar to the conventional mirror-MOTs, with the hybrid-MOT obtaining a greater atom number of the two traps investigated. The vortex-MOT trapped nearly identical number of atoms as the S-MOT, but with lower temperatures due to the continuous cooling force. For the hybrid-MOT the balance between the Zeeman and the spatially variable optical force is close to theoretical optimum and within uncertainty. Differences are due to the difficulty in aligning beams to such high accuracy and also due to imperfect beam intensity balance and nulling of residual magnetic fields. This may have contributed towards the higher temperature, although we believe there may also be optical pumping effects preventing efficient sub-Doppler cooling. What is interesting is the nearly order of magnitude increase of atoms trapped in the hybrid-MOT compared to the vortex-MOT. This cannot be attributed to significantly larger beam overlap volume, nor any other variable which typically result in greater trap loading. The mechanism for this is still unclear and is under further investigation. It should be possible to trap more atoms with more cooling beam power but unfortunately we are limited to the reported maximum beam power.

In summary, we have successfully demonstrated two simplified mirror-MOTs with misaligned (displaced) beam geometries which are suitable for use with microfabricated atom chips and any vacuum chambers with restricted optical access. The design is amenable to microfabrication due to the absence of out-of-plane wires or coils and does not use any frequency selective optics, permitting it to trap different atomic species simultaneously. We have shown the trap parameters closely agree with theoretical predictions and both traps produce comparable number of atoms, as well as temperatures, to standard magneto optical traps. These new MOT designs and techniques will enable to build a portable and miniaturized atom chips for quantum sensors, in addition to finding applications in quantum optics and information.
